# Fluorescent in situ hybridization has limitations in screening NRG1 gene rearrangements

**DOI:** 10.1186/s13000-023-01424-7

**Published:** 2024-01-03

**Authors:** Xiaomei Zhang, Lin Li, Fuping Gao, Binbin Liu, Jing Li, Shuang Ren, Shuangshuang Peng, Wei Qiu, Xiaohong Pu, Qing Ye

**Affiliations:** 1 Department of Pathology, Nanjing Jiangning Hospital, Nanjing, 211100 Jiangsu Province China; 2 Department of Pathology, The Affiliated Drum Tower Hospital of Medical School,Nanjing University, Nanjing, 210008 Jiangsu Province China; 3 Department of Pathology, Nanjing Gaochun People’s Hospital, Nanjing, 210008 Jiangsu Province China; 4 Department of Pathology, Affiliated Hospital of Integrated Traditional Chinese and Western Medicine, Nanjing University of Chinese Medicine, Nanjing, 210008 Jiangsu Province China; 5 Jiangsu Province Academy of Traditional Chinese Medicine, Nanjing, 210008 Jiangsu Province China; 6 Berry Oncology Corporation, Beijing, 100102 China; 7 Department of Pathology, Division of Life Sciences and Medicine, The First Affiliated Hospital of University of Science and Technology of China (USTC), University of Science and Technology of China, Hefei, 230036 Anhui Province China; 8 Intelligent Pathology Institute, Division of Life Sciences and Medicine, University of Science and Technology of China, Hefei, 230036 Anhui Province China

**Keywords:** NRG1, Fluorescence in situ hybridization, Next generation sequencing

## Abstract

**Background:**

NRG1 fusion is a promising therapeutic target for various tumors but its prevalence is extremely low, and there are no standardized testing algorithms for genetic assessment.

**Mothods:**

In this study, we analyzed 3008 tumors using Fluorescence in situ hybridization (FISH) and immunohistochemistry (IHC) to screen for NRG1 translocation and p-HER3 expression.

**Results:**

Our results demonstrated no cases with p-HER3 positivity through IHC. Nonetheless, 29 cases (0.96%) were identified positive for NRG1 translocation through FISH, with three different signal types. FISH-positive cases were subsequently subjected to next-generation sequencing (NGS) testing. However, only eight of these cases were confirmed with NRG1 fusion through NGS. Notably, we divided FISH into three types and FISH type C group was consistent with NGS results. All NGS NRG1 fusion tumors were adenocarcinomas, with a higher prevalence in females. Our findings indicate that although FISH has limitations in screening NRG1 gene rearrangements, NRG1 fusions can be reliably detected with signals exhibiting low copy numbers of the 5’-end of the gene and no fusion signals.

**Conclusion:**

Considering the high cost of NGS, FISH remains a useful method for screening NRG1 fusions in various types of tumors. This study provides valuable insights into the molecular mechanisms of NRG1 fusion and identifies potential treatment targets for patients suffering from this disease.

## Introduction

NRG1 is a member of the neuregulin (NRG) complex family, which is composed of six structurally related cellular growth factors encoded by six closely related genes (NRG1-NRG6). All six NRG family members share a core epidermal growth factor (EGF)-like domain of approximately 65 amino acids and play essential roles in the development of the nervous and cardiovascular systems [1–6]. The EGF-like domain binds to the HER RTK family members (EGFR, HER2, HER3, and HER4) and is fundamental in all NRG members [7]. The NRG1 gene consists of tissue-specific N-terminal exons, immunoglobulin-like (Ig-like) domains, and a common EGF-like domain, and codes for at least 15 different isoforms via alternative splicing, including four heregulin (HRG) isoforms. The binding of HRG ligands to HER3 facilitates heterodimerization of HER3 with HER2 and stimulates a signaling cascade affecting proliferation, survival, and differentiation [8]. NRG1 rearrangement was initially reported in a breast cancer cell line [9] and in 2014, NRG1 gene fusions were identified as activating genomic alterations in invasive mucinous adenocarcinomas of the lung [10]. Recently, NRG1 rearrangement has been identified in several tumors. Although NRG1 gene rearrangement is rare (~ 0.1–0.3%) [11], it has been reported as a potentially actionable genomic event observed in various tumor types. NRG1 fusions can promote pathological signaling via MAPK and other canonical pathways when present [10].Thus, targeting ERBB2 and ERBB3 has been an effective treatment strategy in vitro. Clinical responses to tyrosine kinase inhibitors and mAb have also been recently reported [12–15].

In this study, we systematically gathered an assortment of tumor specimens with the objective of documenting the frequency of NRG1 rearrangements across diverse cancer types. Our aim was to provide a comprehensive characterization of these rearrangements and establish an optimal genetic testing algorithm utilizing various testing menthodologies. Additionally, we sought to generate robust data to inform clinical treatment strategies, leveraging extensive molecular profiling.

## Materials and methods

### Patient data

From September 2015 to December 2021, twelve tumor types including lung cancer(n = 307), gastric cancer(n = 560), breast cancer(n = 141), bladder cancer(n = 154), colorectal cancer(n = 930), liver cancer(n = 108), endometrial cancer(n = 25), cholangiocarcinoma(n = 301), pancreatic cancer(n = 124), laryngocarcinoma(n = 114), esophageal cancer(n = 121) and soft tissue tumor(n = 123) were included in this study. All histologic characteristics were reviewed by a board-certified pathologist. In total, 3008 tumors from pancancer underwent further molecular testing.

### Tissue microarray construction

Each tissue sample underwent immediate fixation in 10% neutral buffered formalin for a duration of 12–48 h, followed by paraffin embedding. The processed samples were subjected to routine deparaffinization and rehydration procedures. A tissue microarray (TMA) was created using the Grand Master automated arrayer (3DHISTECH Ltd., Budapest, Hungary), with 2 mm punch size obtained from representative tumor blocks of each case. The tumor core was extracted from the invasive front of the deepest tumor invasion portion, with avoidance of necrotic areas. Dual representations of TMAs were constructed and then sectioned into 4-µm-thick sections for histological, immunohistochemical, and FISH detection procedures. IHC and FISH were performed in both duplicates of each case, and in case of disagreement between the duplicates in FISH or IHC results, the entire slide was used for a final decision.

### Fluorescence in situ hybridization (FISH)

**Fig. 1:**
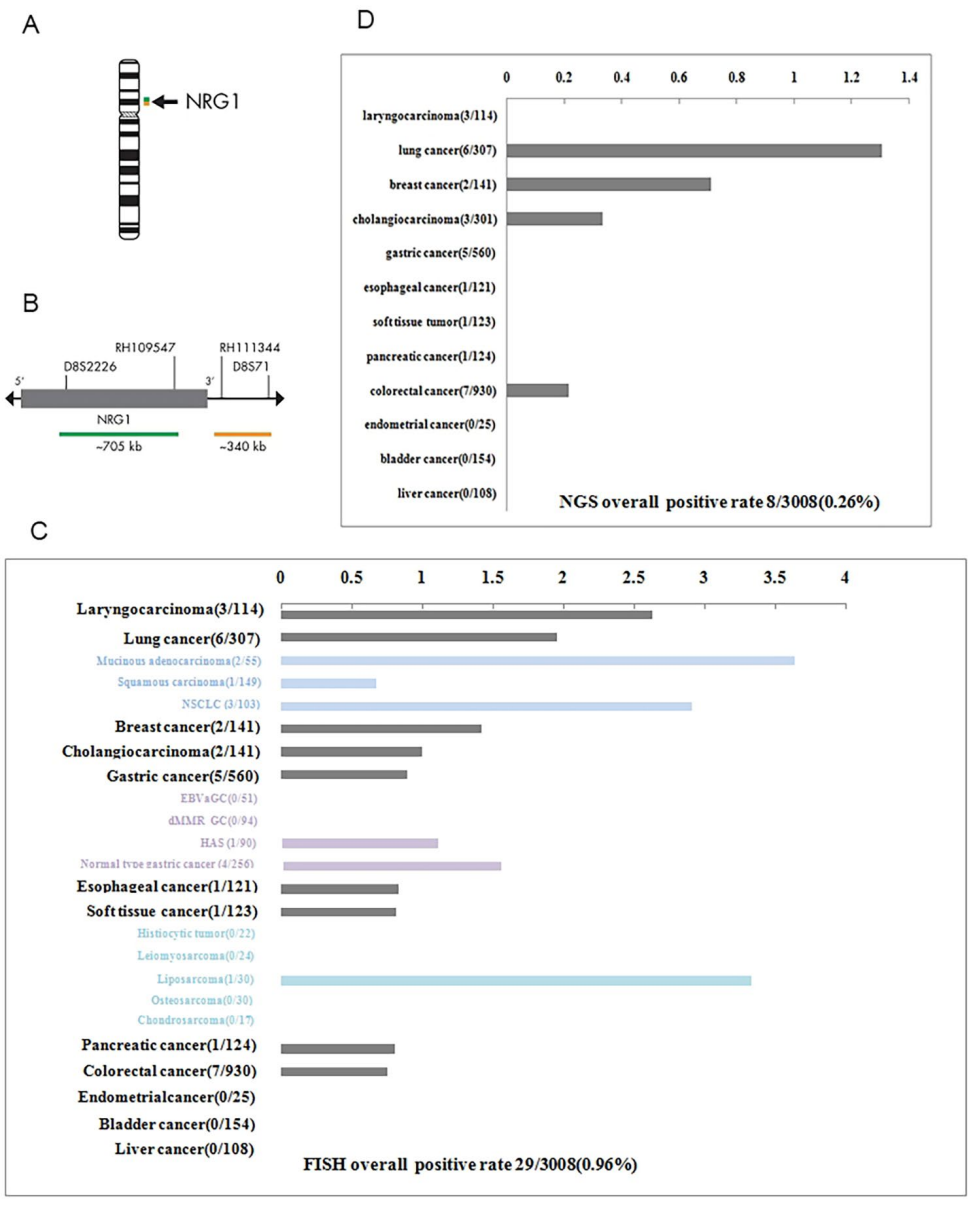
NRG1 fusion prevalence across cancers. **A** The NRG1 dual color break-apart probe is designed to detect translocations involving the chromosomal region 8p12 harboring the NRG1 gene. **B** Two-color FISH probes flanking the 5’-end (green) and the 30-end (orange) of the NRG1 gene were performed on the 3008 cases. **C** NRG1 translocation-positive cases by FISH in 3008 pancancers. **D** NRG1 fusion-positive cases by NGS in 3008 pancancers

Four-micrometer-thick, formalin-fixed and paraffin-embedded tissue sections were used for FISH. FISH testing for NRG1 gene rearrangements was performed using the NRG1 Dual Color Break Apart Probe (ZytoVision, Germany). Probes used the following BACs from the Human BAC Library: BACs D8S2226 and RH109547 were labelled with Spectrum Green (5’ terminus of NRG1), and BACs RH111344 and D8S71 were directly labelled with Spectrum Orange (3’ terminus of NRG1). NRG1 gene break-apart **(**Fig. 1A and B**)** was performed according to the operating instructions. In our study, FISH positivity was defined when more than 15% of tumor cells displayed signals with distinct red and green signals or a signal pattern that maintained a single red signal.

### Immunohistochemistry (IHC)

p-ERBB3 (Clone: Tyr1289, Cell Signaling, USA) IHC staining was carried out on an automatic Ventana Bench Mark Ultra system (Roche Diagnostics, Basel, Switzerland) using an automated staining protocol. IHC staining scores were calculated by multiplying the staining intensity (0 = no staining, 1 = mild staining, 2 = moderate staining, and 3 = strong staining) by the percentage of immunoreactive tumor cells (0 to 100). The immunostaining result was considered to be 0 or negative when the score was < 25; 1 + or weak when the score was 26–100; 2 + or moderate when the score was 101–200; or 3 + or strong when the score was 201–300. The IHC results were interpreted independently by two pathologists who were blinded to all clinical and pathological data. p-ERBB3 IHC was regarded positive when IHC staining score > 0.

### DNA/RNA extraction

Genomic DNA (gDNA) and total RNA were extracted from formalin-fixed paraffin-embedded (FFPE) tumor tissue samples via the Prep DNA/RNAFFPE Kit (Qiagen, USA) based on the manufacturer’s instructions. The quality of purified DNA/RNA was analysed by gel electrophoresis and quantified by Qubit® 4.0 Fluorometer (Life Technologies, USA). An amount of extracted DNA greater than 30 ng was considered sufficient for analysis. In the extracted FFPE RNA samples, the 28 S and 18 S rRNA bands were degraded, and ≥ 200 ng RNA was optimal for high analytical sensitivity.

### DNA-based NGS

First, the above purified gDNA was fragmented into DNA pieces using an enzymatic method (5* WGS Fragmentation Mix, Qiagen, USA), followed by end repair, T-adaptor ligation, and PCR amplification, resulting in a prelibrary. The DNA was then subjected to 654 cancer-related gene target capture using the Solid Tumor ComprehensiveTest Kit (Berry Oncology Corporation) to detect SNV/Indel, CNV and gene fusions.

Sequencing libraries were generated after PCR amplification and then sequenced on a NovaSeq 6000 platform (Illumina, San Diego, USA) with 150PE mode. Alignment against the human reference genome hg19 was performed using BWA [16]. SAM tools [17] and the Genome Analysis Tool kit GATK 3.8 [18] were utilized to call SNVs and small indel variants. Large indels and chromosomal rearrangements (including NRG1 rearrangements) were analysed using Fusion map [19]. Fusions with a supported mutation fragment number ≥ 2 were identified and reported. For breakpoints in intergenic regions, the nearest gene in each direction was reported as the predicted fusion partner.

### RNA-based NGS

An in-house designed RNA fusion panel based on hybrid capture sequencing (Berry Oncology Corporation) was performed to detect gene fusions, which tiled all exons of general fusion genes in tumors and allowed for the detection of known and novel fusions. Briefly, the purified total RNA was first converted to complimentary DNA through reverse transcription. The prelibrary construction consisted of end repair, adaptor ligation and PCR amplification. The following hybridization-captured libraries were sequenced on a NovaSeq 6000 platform (Illumina, San Diego, USA) in 150PE mode. Gene fusions were called based on Fusion map software [19]. Additionally, these fusions identified via bioinformatics were verified by manual inspection of the breakpoints. Both DNA and RNA based NGS positivity were defined as the detected rearrangement abundance (AF) being greater than 5%.

To avoid the heterogeneity of tumors, we selected the entire tumor sample for both DNA-based NGS and RNA-based NGS testing.

## Results

### Sample population

From September 2015 to December 2018, a total of 3008 distinct tumor specimens from patients were evaluated. The cohort encompassed various tumor types, including lung cancer (n = 307), gastric cancer (n = 560), breast cancer (n = 141), colorectal cancer (n = 930), liver cancer (n = 108), bladder cancer (n = 154), endometrial cancer (n = 25), cholangiocarcinoma (n = 301), pancreatic cancer (n = 124), laryngocarcinoma (n = 114), esophageal cancer (n = 121), and soft tissue tumors (n = 123). Each tumor type was further subdivided as follows: lung cancer included non-small cell lung carcinoma (n = 103), squamous carcinoma (n = 149), and mucinous adenocarcinoma (n = 55); within gastric cancer, subgroups comprised Epstein-Barr virus-associated gastric cancer (n = 51), DNA mismatch repair protein-deficient gastric cancer (n = 94), hepatoid adenocarcinoma (n = 90), and normal type of gastric cancer (n = 256). Breast cancer was categorized into triple-negative (n = 53) and non-triple-negative (n = 88) subtypes, while all 930 recorded cases of colorectal cancer were classified as adenocarcinomas. All 301 assessed cholangiocarcinomas were intrahepatic. Finally, soft tissue tumors were subtype-specific, including malignant fibrous histiocytic tumors (n = 22), leiomyosarcomas (n = 24), liposarcomas (n = 30), osteosarcomas (n = 30), and chondrosarcomas (n = 17).

### Three distinct FISH signal types of NRG1 aberrations

**Fig. 2:**
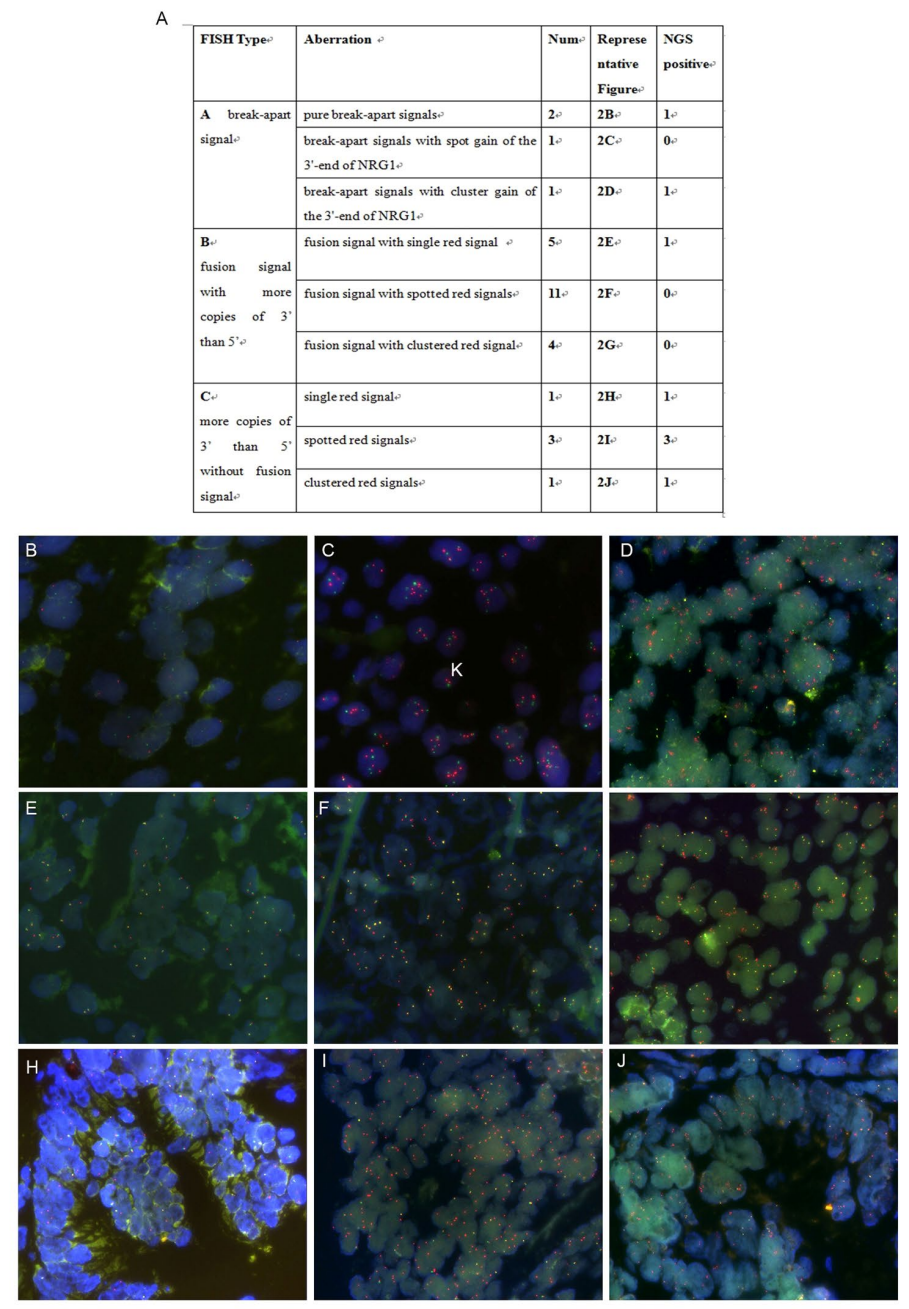
Distinct FISH signal types of NRG1 aberrations. **A** Summary of NRG1 aberrations as determined by FISH. **B-J** Microscopic images demonstrating examples of the three types of NRG1 aberrations as determined by FISH. Green spots represent the 5’ end of the NRG1 gene, and red spots represent the 3’ end of the NRG1 gene

The NRG1 dual color break-apart probe was specifically designed to identify the occurrence of translocations within the chromosomal region 8p12 that contains the NRG1 gene (Fig. 1A). The green probe covered NRG1 exons 1–2 and the chromosomal region preceding the 5’ end of the NRG1 gene, while the orange probe was responsible for identifying the chromosomal region following the 3’ end of the NRG1 gene (Fig. 1B). Breakpoints within the NRG1 gene resulted in translocation events that retain the 3’ end of NRG1 and consequently, only signals displaying separate red and green signals or the signal pattern that retains the single red are considered indicative of NRG1 rearrangement [20]. Out of the 3008 cases evaluated, 29 cases (0.96%) showed abnormal NRG1 signals, not taking into account any potential instances of aneuploidy. The incidence of NRG1 abnormalities varied depending on the type of tumor examined (Fig. 1C), with a rate of 1.95% in lung cancer (6/307), 0.89% in gastric cancer(5/560), 1.42% in breast cancer (2/141), 0.75% in colorectal cancer (7/930), 1.00% in cholangiocarcinoma (3/301), 0.81% in pancreatic cancer (1/124), 2.63% in laryngocarcinoma (3/114), 0.83% in esophageal cancer (1/121), and 0.81% in soft tissue tumors (1/123). With respect to FISH signals, three distinct types of abnormalities were observed: (A) break-apart signal (three cases) with or without high copy number of the 3’-end of the gene; (B) low copy number of the 5’-end of the gene with respect to the 3’-end of the gene, with fusion signals (12 cases); and (C) low copy number of the 5’-end of the gene with respect to the 3’-end of the gene, without fusion signals (Fig. 2A). Type A aberrations were rarely observed throughout this study. The four cases exhibiting type A NRG1 aberrations presented purely break-apart signals, while the other two cases manifested spot or cluster gain of the 3’-end of NRG1 along with a break-apart signal (Fig. 2B and D). Type B aberrations were the most commonly observed within this study. All type B cases featured a single tumor cell remaining as one or more fusion cells, indicating the presence of at least one copy of NRG1. In addition to the fusion signal, there were also additional single dots or clusters of red signals (Fig. 2E and G). All such cases displayed partial loss of the 5’-end and gain or amplification of the 3’-end of NRG1. The remaining five cases with type C abnormalities displayed increased copy number of the 3’-end of NRG1 triggered by the loss of the 5’-end of the gene without any fusion signals (Fig. 2H J).

### Genome rearrangements were not fully associated with NRG1 fusions

**Fig. 3:**
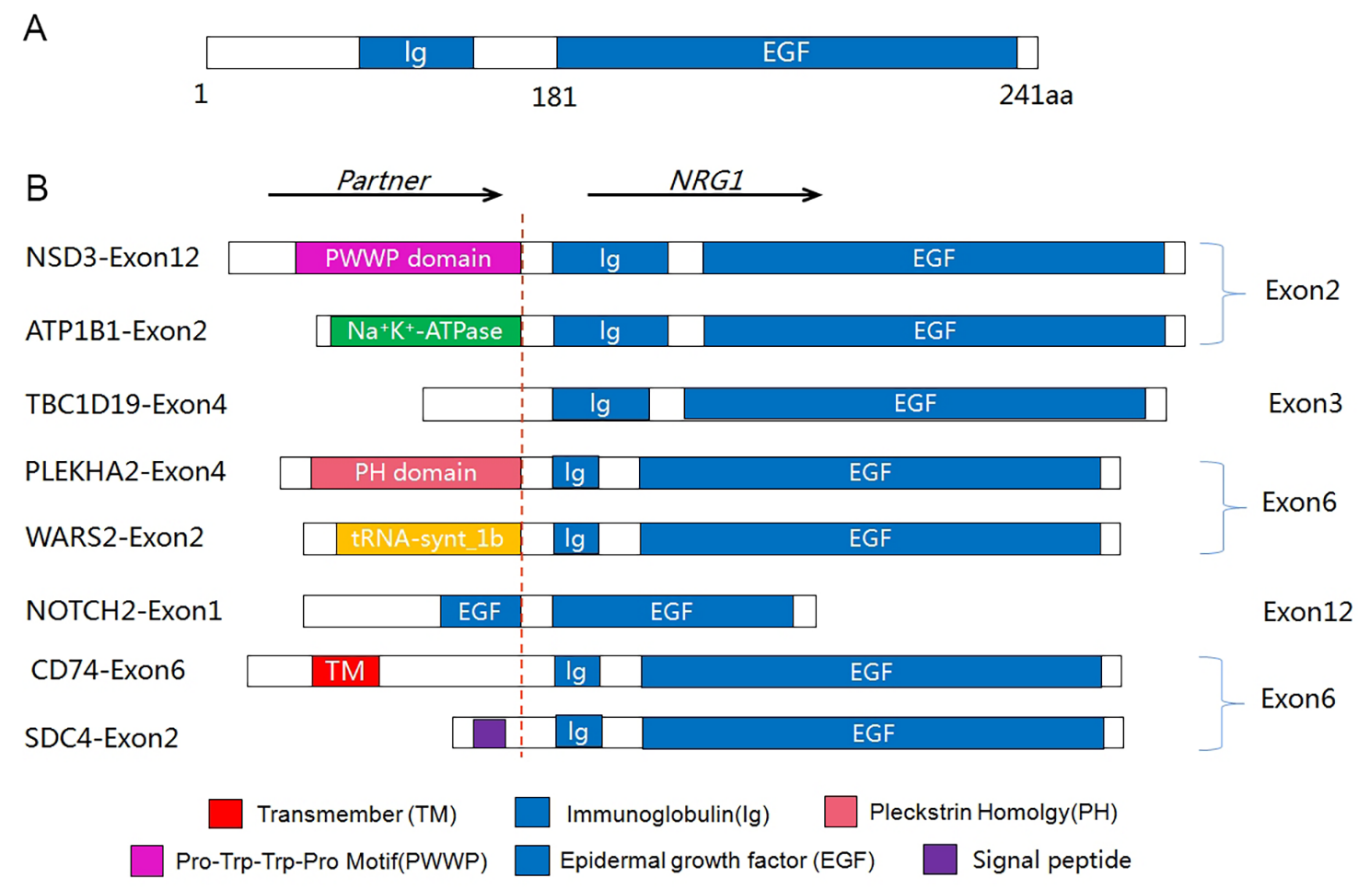
Schematic diagram of NRG1 fusion variants in pancancers. **A** Genomic structure of wild-type NRG1. **B** Fusion variants identified with 5’ partners joined to 3’ NRG1. Bars depict the predicted functional domains (not shown to scale) of interest, and the red dashed line indicates fusion breakpoints. The EGF domain is preserved in all fusion variants

In order to validate the FISH results, all of the FISH-positive cases underwent further testing using NGS (both DNA- and RNA-based). However, the results of the FISH analysis were not in agreement with the NGS results. Out of the 29 cases with abnormal NRG1 FISH signals, only eight cases were conclusively proven to have NRG1 fusion through the use of NGS (Fig. 1D). Break-apart signals or fusion signals possessing more copies of 3’ than 5’ NRG1 do not necessarily indicate the presence of true fusions. Nevertheless, FISH results indicated that the presence of more copies of 3’ than 5’, without a fusion signal, was linked to NRG1 fusion. All four of the cases with FISH type C signals were confirmed to be NRG1 fusions through the use of both DNA- and RNA-based NGS techniques. A schematic representation of the eight NRG1 jfusions is displayed in Fig. 3. Breakpoints were most commonly observed after exon 6, followed by exon 2, exon 3, and exon 12. Of the eight cases featuring NRG1 fusions, two were lung adenocarcinoma, two were mucinous adenocarcinoma of the lung, two were colorectal adenocarcinoma, one was intrahepatic cholangiocarcinoma, and one was breast invasive carcinoma, thus exhibiting a predominance of lung cancer. A detailed summary of the clinical, pathological, immunological, and molecular features of the eight NRG1 fusion-positive cases may be found in Table 1. Notably, tumors harboring an NRG1 fusion all presented histologically as adenocarcinomas and occurred primarily in female patients with only one male patient, thereby exhibiting a female predominance.

**Table 1:** Clinicopathological features of eight NGS NRG1 fusion positive cases

No.	Tumor type	Histological type	Gender	Age	Stage(T,N,M)	NRG1 fusion mode
S201922867-3	colorectal cancer	Adenocarcinoma	Male	78	II(T3,N0,M0)	NSD3:EXON3-NRG1:EXON 2
S201906567-2	colorectal cancer	Adenocarcinoma	Female	56	II(T2,N0,M0)	WASR2:EXON2-NRG1:EXON6
S202022206	Intrahepatic cholangiocarcinoma	Adenocarcinoma	Female	39	I(T1,N0,M0)	ATP1B1:EXON2-NRG1: EXON2
201766478	Breast cancer	Invasive adenocarcinoma	Female	51	III(T2,N2,M0)	PLEKHA2:EXON4-NRG1: EXON6
201770699-3	Lung cancer	NSCLC	Female	46	IV(T2,N0,M1)	NOTCH2:EXON1-NTR1:EXON12
F201900641-4	Lung cancer	NSCLC	Female	65	II(T1,N2,M0)	TBC1019:EXON4-NRG1:EXON3
JF201900557	Lung cancer	Mucinous adenocarcinoma	Female	67	I(T1,N0,M0)	CD74:EXON6-NRG1:EXON 6
JF202001074	Lung cancer	Mucinous adenocarcinoma	Female	70	I(T1,N0,M0)	SDC4:EXON2-NRG1:EXON 6

### Phospho-ErbB3 and Phospho-ErbB2 IHC is useless for NRG1 fusion prediction

It has been reported that the presence of NRG1 rearrangements results in an increase in fusion transcript and chimeric ligand, leading to aberrant induction of ErbB2/ErbB3 heterodimerization and subsequent receptor activation by phosphorylation. Previous studies have demonstrated that the phospho-ErbB3 immunohistochemistry (IHC) assay exhibits a sensitivity of 100% and specificity of 97% for identifying cases with NRG1 rearrangements [21]. However, our study revealed that all 29 cases that were positive by fluorescence in situ hybridization (FISH) for NRG1 rearrangements were negative for phospho-ErbB3 IHC staining, and even among the eight cases that were positive by next-generation sequencing (NGS), none exhibited any staining. Based on these findings, we conclude that phospho-ErbB3 is not a useful biomarker for screening NRG1 fusion in our study cohort.

## Discussion

The present study investigated 3008 specimens from various tumor types to identify NRG1 fusions. Initially, 29 cases of NRG1 translocations were identified by fluorescence in situ hybridization assay in multiple cancer types. However, further confirmation by next-generation sequencing revealed a significantly lower incidence of NRG1 fusion, ultimately identifying eight NRG1 fusion-positive specimens. Specifically, this molecular alteration was observed in adenocarcinomas derived from lung, breast, cholangiocarcinoma, and colorectal tissues, with an overall low incidence. The majority of NRG1 fusion tumors (87.5%) were observed in female patients. The breakpoint commonly occurred after exon 6, followed by exon 2, 3, and 12. Furthermore, eight different fusion partners were noted in the positive cases. In lung cancer, the NRG1 fusion has more frequently been observed in the invasive mucinous adenocarcinoma subtype, and in this study, half of the NRG1-positive non-small cell lung cancer cases demonstrated a mucinous histology. In breast cancer, the incidence of NRG1 fusion was reported to be 0.35%, with an associated adverse prognosis [22, 23]. This study identified only one case of PLEKHA2-NRG1 fusion with an incidence of 0.71% in breast cancer cases. Pancreatic ductal adenocarcinoma (PDAC) has been reported to be an NRG1 fusion-enriched tumor; the overall incidence of NRG1 + PDAC is estimated to be 0.48% [11], and the incidence of NRG1 + PDAC seems to be enriched in KRAS-wild-type PDAC [24, 25]. However, in this study, we did not find NRG1 fusion in pancreatic cancer, mostly because of the limited samples. Remarkably, colorectal cancer and cholangiocarcinoma exhibited a relatively higher incidence of NRG1 fusion (0.22% and 0.33%, respectively). The limited data pertaining to a predominant histology, molecular subgroup, or hormonal status associated with NRG1 fusion in specific tumor types necessitates further investigation in terms of effective screening strategies.

The optimal methodology for screening NRG1 fusions across various cancers remains unclear. Break-apart FISH is a commonly employed technique for detecting fusions, and it has been used since 2004 to detect NRG1 fusions. In a previous study by Prentice et al., NRG1 rearrangements were found in 17 out of 382 breast cancer cases. Three types of signal aberrations were observed, including amplification of either the 5’ or 3’ ends, or both. However, a novel amplicon centromeric to NRG1 was discovered through bacterial artificial chromosome array comparative genomic hybridization, which encompassed two genes (SPFH2 and FLJ14299). It was suspected that this amplicon resulted from breakpoints and chromosomal rearrangements within the NRG1 locus [23]. Similar to Prentice’s study, our study also observed three types of NRG1 break-apart signal aberrations. However, the FISH probe design in both studies did not specifically target the NRG1 gene, which may have contributed to false-positive results. Furthermore, the complexity of the rearrangement mechanism, such as break-fusion-bridge (BFB) cycles and out-of-frame variants, can also lead to deceptive gene rearrangements. BFB cycles involve breakpoints surrounding the amplicon and loss of genes telomeric to the amplified region, which can result in amplified regions centromeric to the NRG1 breakpoint and overexpression of novel oncogenes [26]. A similar scenario in which these breakpoints result in break-fusion-bridge (BFB) cycles that create amplified regions centromeric to the target, leading to overexpression of novel oncogenes, has been suggested for Her2, Topo2A and RET [27, 28]. Out-of-frame (nonfunctional) variants are also denoted as NRG1 rearrangements, which can also induce false-positive FISH results but negative RNA-based NGS results. RNA-based NGS has the advantage of providing evidence that a putative fusion is expressed and in-frame, as not all RNA transcripts are in-frame. However, DNA-based sequencing cannot capture transcribed products when there are multiple splice variants and transcriptional start sites, so DNA-based sequencing can only predict a transcript but cannot guarantee an in-frame messenger RNA product [29, 30]. Therefore, RNA-based NGS is advantageous in detecting true NRG1 fusions by providing evidence of expressed and in-frame fusion transcripts. Additionally, another advantage of RNA-based NGS is that it can calculate both the ratio of β/α-isoforms and the frequency of NRG1 fusions from the numbers of junctional reads, including the ratio of β/α-isoforms [31]. However, limitations exist due to the difficulty of obtaining RNA of sufficient quality and quantity from clinical samples, especially those preserved in formalin-fixed, paraffin-embedded tissues. In conclusion, the strategy for screening NRG1 fusions in cancers is complex and requires further investigation.

NRG1 gene undergoes alternative splicing to produce various heregulin isoforms, which act as ligands for members of the HER/ERBB receptor family, particularly HER2 and HER3 [7, 32]. As a result, phosphorylated HER3 (p-HER3) is commonly used as a functional screen for NRG1 fusions. Immunohistochemical (IHC) detection of p-HER3 has shown sensitivity rates of 94–100% [10, 21]. However, in our study, we did not detect any cases positive for p-HER3 IHC. Limitations associated with IHC testing include the potential for both false negative and false positive results. Factors contributing to false negative results include variations in tissue fixation conditions and the suboptimal sensitivity of p-HER3 antibodies. False positive results may arise from the expression of tissue-restricted NRG1 isoforms and the intrinsic presence of NRG1 in certain neural crest-derived tissues. Therefore, further investigations are necessary to establish the applicability of p-HER3 IHC screening across various tissues and tumor types.

In this study, we investigated NRG1 fusion in a large pancancer cohort using NGS and FISH techniques. Our findings revealed that the prevalence of NRG1 fusion was significantly lower than previously anticipated, with only eight specimens demonstrating such fusions through NGS analysis (8/3008, 0.27%). Breakpoints were seen most frequently to occur after exon 6, followed by exons 2, 3, and 12, with diverse fusion partners observed. Clinically, all NRG1 fusion tumors were adenocarcinomas, with the majority registered as female (7/8, 87.5%). Interestingly, NRG1 fusion was also detected in cholangiocarcinoma and colorectal carcinoma, besides the well-known breast and lung cancers. Of the total cases, 29 were FISH positive, but only eight were confirmed via NGS analysis. We believe that FISH analysis may be deceptive in identifying NRG1 gene rearrangements, potentially due to BFB cycles induced by breakpoints within the NRG1 gene, resulting in the amplification of novel oncogenes. Nevertheless, Type C FISH cases were found to be in complete accordance with NGS results.
